# Traffic Flow Prediction in 5G-Enabled Intelligent Transportation Systems Using Parameter Optimization and Adaptive Model Selection

**DOI:** 10.3390/s24206529

**Published:** 2024-10-10

**Authors:** Hanh Hong-Phuc Vo, Thuan Minh Nguyen, Khoi Anh Bui, Myungsik Yoo

**Affiliations:** 1 Department of Electronic Engineering, Soongsil University, Seoul 06978, Republic of Korea; hanhvo@soongsil.ac.kr (H.H.-P.V.); 1102360003@soongsil.ac.kr (T.M.N.); kbui@soongsil.ac.kr (K.A.B.); 2 School of Electronic Engineering, Soongsil University, Seoul 06978, Republic of Korea

**Keywords:** traffic flow, parameter optimization, whale optimization algorithm, genetic algorithm, fast variation mode decomposition

## Abstract

This study proposes a novel hybrid method, FVMD-WOA-GA, for enhancing traffic flow prediction in 5G-enabled intelligent transportation systems. The method integrates fast variational mode decomposition (FVMD) with optimization techniques, namely, the whale optimization algorithm (WOA) and genetic algorithm (GA), to improve the accuracy of overall traffic flow based on models tailored for each decomposed sub-sequence. The selected predictive models—long short-term memory (LSTM), bidirectional LSTM (BiLSTM), gated recurrent unit (GRU), and bidirectional GRU (BiGRU)—were considered to capture diverse temporal dependencies in traffic data. This research explored a multi-stage approach, where the decomposition, optimization, and selection of models are performed systematically to improve prediction performance. Experimental validation on two real-world traffic datasets further underscores the method’s efficacy, achieving root mean squared errors (RMSEs) of 152.43 and 7.91 on the respective datasets, which marks improvements of 3.44% and 12.87% compared to the existing methods. These results highlight the ability of the FVMD-WOA-GA approach to improve prediction accuracy significantly, reduce inference time, enhance system adaptability, and contribute to more efficient traffic management.

## 1. Introduction

The surge in traffic volume has exacerbated congestion, placing immense pressure on transportation infrastructure. Addressing these challenges requires innovative approaches to forecasting traffic patterns and efficiently managing the flow of vehicles in complex urban environments. In this context, 5G technology plays a pivotal role by offering high-speed, low-latency communication, which allows for real-time data collection of large volumes of traffic data from various sensors, cameras, and connected vehicles. This means traffic data can be collected instantly, making real-time monitoring more accurate and timely. This wealth of real-time data enables the development of intelligent transportation systems (ITS) that can enhance traffic flow prediction to contribute to more efficient traffic management. Furthermore, 5G technology also helps deploy traffic flow prediction models based on machine learning to process in real-time through edge computing.

Despite the potential of 5G-enabled ITS, traffic flow prediction remains a challenging task due to the non-stationary nature of traffic data, which are influenced by a variety of factors, including weather conditions, human activities, and unforeseen events [1]. These factors introduce fluctuations that reduce prediction accuracy. Therefore, preprocessing is essential to ensure satisfactory prediction accuracy. Techniques for processing time-series data—including decomposition—are commonly used for this purpose, with popular methods including wavelet decomposition, empirical mode decomposition (EMD), and variational mode decomposition (VMD). Wavelet decomposition splits data into different frequency modes to extract traffic flow information; however, selecting an appropriate wavelet basis can be challenging [2,3,4]. EMD adaptively decomposes data to enhance predictive performance without requiring a selection of the number of modes; however, it can cause endpoint effects and mode mixing [5,6,7]. VMD, introduced by Dragomiretskiy in 2014, is a nonrecursive, adaptive, and quasi-orthogonal method [8]. Several of the prior studies [9,10,11] have also shown that VMD effectively solves modal aliasing and improves traffic flow prediction accuracy.

When utilizing VMD, the selection of appropriate values K and the α parameter is pivotal, as these values significantly affect the accuracy of forecast outcomes [10,12,13]. Parameter K controls the number of output sub-sequences of the VMD, while alpha is the moderate bandwidth penalty constraint that controls the smoothness of each sub-sequence. A higher value of α results in more narrow subsequences and is less prone to capturing noise. The previous studies have employed optimization algorithms, such as genetic algorithms (GA) [14,15], whale optimization algorithm (WOA) [16,17], and particle swarm optimization (PSO) [18,19], to fine-tune these parameters.

In addition, the original VMD framework is typically solved using the alternating direction method of multipliers (ADMM), which is a popular technique for distributed optimization owing to its numerous advantages, including a modular structure, straightforward implementation, excellent convergence, and flexibility [8]. Although ADMM has a convergence rate of O(1/k), it might not be sufficiently quick in complex scenarios, resulting in time-consuming operations for the entire system [20]. Furthermore, optimization-based VMD is known to be highly time-consuming, as mentioned in [21,22]. Thus, the acceleration of VMD can lead to a decrease in resource usage and an improvement in overall performance.

Moreover, because the output of VMD consists of K subsequences with different features, the use of different models for these subsequences can improve the predictive performance [23]. However, the prior studies did not address the issue of parameter optimization for FVMD and model selection for each subsequence simultaneously, thereby compromising model flexibility. Instead, most of the prior studies adopted a single model for all subsequent data, ignoring differences in the characteristics of subsequences [2,24,25].

These problems motivated us to propose a novel system for predicting the traffic flow for intelligent transportation systems integrated with 5G technology. To the best of our knowledge, this study represents the first attempt to improve VMD speed and use WOA-GA to fine-tune VMD parameters, enabling the selection of a suitable model for each subsequence after decomposition. The main contributions of this study are summarized as follows:

1. We introduce FVMD as a novel VMD variant that employs fast ADMM instead of ADMM to enhance its convergence rate.
2. We also propose the hybrid WOA-GA to fine-tune FVMD parameters, enabling the selection of a suitable model for each subsequence based on its unique characteristics.
3. We conducted experiments to evaluate the effectiveness of traffic flow prediction using several metrics—including the mean square error (MSE), root mean square error (RMSE), mean absolute error (MAE), and mean absolute percentage error (MAPE)—on two real-world datasets.

The paper is organized as follows: Section 2 surveys the existing research on traffic flow prediction in 5G-enabled intelligent transportation systems. Section 3 outlines the fundamental techniques employed in the proposed approach. Section 4 presents the evaluation results, and Section 5 concludes the paper.

## 2. Related Work

This section examines prior studies associated with traffic volume decomposition using VMD. Table 1 presents a summary of the related works.

Hong Yang et al. [26] integrated cosine similarity (CS)-based VMD (CSVMD) with an extreme learning machine (ELM) and an iterative error compensation strategy to create a new model called CSVMD-ELM-error. This model was designed to address the limitations of the existing traffic flow data prediction models, particularly those related to mode mixing and the selection of mode numbers. The authors decomposed traffic flow data into intrinsic mode functions (IMFs) using VMD and then employed ELM to predict each IMF. In addition, they determined the optimal mode number (K) for VMD by calculating the CS of the original and decomposed data. As the K value increased, the CS value continuously increased until it reached the optimal K value, i.e., the K value closest to 1. The CSVMD-ELM-error model demonstrated superior prediction performance for various traffic flow data datasets.

Huang et al. [25] investigated the effects of various multi-scale decomposition algorithms on neural network performance, specifically in the context of short-term traffic flow prediction. These algorithms included empirical mode decomposition (EMD), ensemble EMD (EEMD), complete EEMD with adaptive noise (CEEMDAN), VMD, wavelet decomposition (WD), and wavelet packet decomposition (WPD). After being decomposed into various components, the traffic flow data were analyzed using a bidirectional long short-term memory (BiLSTM) neural network model. The study provides comprehensive insights into the selection of multiscale decomposition algorithms.

Yin et al. [27] introduced a hybrid model called ST-VGBiGRU, for traffic flow prediction, effectively capturing both spatial and temporal correlations in traffic data. The authors utilized the VMD algorithm to decompose traffic flow sequences into stationary modal components, which were then noise-reduced using fuzzy entropy to enhance accuracy. A hybrid graph attention network–bidirectional gated recurrent unit (GAT-BiGRU) model was applied to each IMF. The GAT network captured the attention levels of prediction nodes to neighboring traffic nodes, whereas the BiGRU network captured the temporal correlation of each modal component. The ST-VGBiGRU model demonstrated significant improvements in prediction accuracy on the RTMC traffic dataset. However, model performance tended to decrease over longer prediction horizons, suggesting that although the model excels in short-term predictions, its effectiveness may diminish in longer-term forecasting scenarios.

Zhong et al. [28] introduced a hybrid model that combines VMD, a deep extreme learning machine (DELM), and Harris hawks optimization (HHO). They used VMD to decompose short-term traffic flows, and combined an extreme learning machine (ELM) with the deep neural network framework of a stacked autoencoder (AE) to construct the DELM, which was subsequently optimized via HHO. The VMD-HHO-DELM model demonstrated significant improvements in prediction accuracy, achieving $R^{2}$ scores of 0.9984 for non-stationary data and 0.9993 for stationary data.

Zhao et al. [9] introduced a short-term traffic flow prediction model that combines VMD with an improved dung beetle optimization (IDBO) algorithm to optimize LSTM networks, effectively addressing the challenges posed by nonlinear traffic flow data characterized by high noise levels. VMD was initially employed to decompose the original traffic flow sequence into several IMFs, and the LSTM networks with hyperparameters optimized using IDBO were subsequently deployed to predict traffic flow based on the decomposed IMFs. The proposed VMD-IDBO-LSTM model demonstrated significant improvements in prediction accuracy compared to the traditional models, including single LSTM and other combined models such as EMD-LSTM. Despite these improvements, the IDBO algorithm still faces challenges related to local optima, which can affect overall prediction accuracy.

Yin et al. [28] developed a new hybrid model for short-term traffic flow forecasting, incorporating VMD, a group method of data handling (GMDH) neural network, BiLSTM, and an ELMAN network optimized by the imperialist competitive algorithm (ICA). Comparative experiments demonstrated that this model exhibits excellent predictive capabilities, with the BiLSTM, GMDH, and ELMAN networks outperforming other single models, and VMD significantly improving predictive performance. Subsequently, Yin et al. confirmed the effectiveness of VMD by combining LSTM with each individual component [30].

Li et al. [10] developed a secondary decomposition system using a complete ensemble empirical mode decomposition with adaptive noise (CEEMDAN), neural network estimation time entropy (NNetEn), and VMD enhanced by the northern goshawk optimization (NGO) algorithm. The kernel extreme learning machine (KELM) was optimized by the artificial rabbits optimization (ARO) algorithm with error correction (EC). High- and low-frequency IMF components were separated using NNetEn following CEEMDAN decomposition. VMD, fine-tuned by NGO, performed a secondary decomposition on all high-frequency components. All decomposed components were then predicted using ARO-KELM, with error correction included to further improve accuracy.

Dai et al. [11] introduced optimized variational mode decomposition (OVMD), where VMD was fine-tuned using the bat algorithm (BA). The predictor for each subsequence combined the basic LSTM network with the BiLSTM network.

Guo et al. [31] proposed a hybrid model that utilizes VMD and autocorrelation for long-term traffic flow prediction, wherein VMD is used to decompose data into intrinsic mode functions and extract features to capture traffic flow changes. They incorporated a correction module with a convolutional layer and used it alongside an autocorrelation mechanism to build an encoder and decoder for feature extraction and fusion.

In general, the effectiveness of traffic flow predictions is enhanced by combining data decomposition methods with prediction models. However, the aforementioned studies failed to address potential tasks that may further improve predictive performance. First, most prior studies did not consider improving the speed of optimization-algorithm-based VMD. Second, they did not consider selecting an appropriate model for specific characteristics; instead, each of the aforementioned studies used a single model for all subsequences after decomposition.

## 3. Proposed Work

### 3.1. Overall Architecture

**Figure 1:**
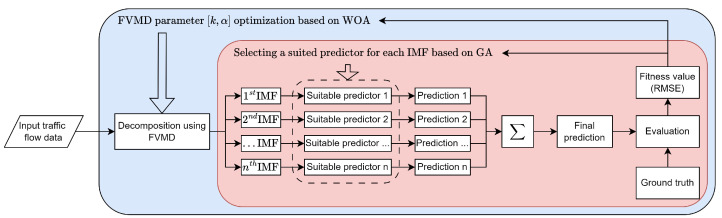
The overall architecture of our approach.

This study proposes a novel approach to enhance the performance of traffic flow data prediction by introducing FVMD and WOA-GA. The overall architecture of the proposed method is illustrated in Figure 1. Specifically, we replaced the ADMM in VMD with fast ADMM to enhance convergence speed, accelerating the decomposition of input traffic flow data to IMFs. Furthermore, the proposed WOA-GA fine-tunes the FVMD parameters, including K and α, and selects a suitable machine learning (ML) model for each IMF. The list of candidate ML models includes the gated recurrent unit (GRU) [32], bidirectional GRU (BiGRU) [33], LSTM [34], and BiLSTM [35]. Finally, the predictions from all branches are fused to generate the final prediction. The RMSE, employed to compare the final prediction to the ground truth, served as the fitness value for WOA-GA.

### 3.2. FVMD Algorithm

VMD is a signal-smoothing technique, wherein nonlinear historical traffic flow data are smoothed and broken down into some bits of comparatively stable data. This is crucial when predicting traffic flow, as predictive performance can be significantly enhanced if the input model contains both smooth and stable data. Most prediction models are not particularly successful in predicting non-stationary data.

The fundamental structure of a VMD is a variational algorithm that partitions nonstationary signals into many intrinsic mode components with a restricted bandwidth. Although the classical ADMM is widely used to resolve this problem, it has a relatively slow convergence rate of O(1/k). As an accelerated variant of ADMM, fast ADMM, employs the Nesterov technique, achieving a faster convergence rate of $O(1/k^{2})$ for strongly convex problems [36]. Our proposed FVMD algorithm employs fast ADMM to improve VMD speed.

(1) **Formulate the variational problem.**

A one-sided spectrum is derived by applying the Hilbert transform to compute the analytical signal for each mode function. The mode spectrum is then transformed into baseband, and the predicted center frequency is determined via exponential adjustment.

(1) $$\min_{u_{k},w_{k}}\left\{\sum_{k=1}^{K}{∥\partial_{t}\left[\left(\delta \left(t\right)+\frac{j}{\pi t}\right)*u_{k}\left(t\right)\right]e^{-jw_{k}^{t}}∥}_{2}^{2}\right\}\;s.t.\sum_{k=1}^{K}u_{k}=f$$

where $u_{k}$ indicates the fractions of the mode produced from signal decomposition, ${\hat{u}}_{k}$ represents the center frequency of these mode fractions, *K* denotes the number of iterations, *k* takes values from 1 to *K*, δ(t) represents the Dirac distribution, * represents the convolution operation, $w_{k}$ represents the central frequency, and *f* denotes the original signal.

(2) **Transform the confined variational problem into an unconstrained variational problem.**

Equation (2) represents the modified Lagrange expression, incorporating a quadratic penalty factor α and Lagrange multiplicative operator λ(t) derived from Equation (1):

(2) $$\begin{array}{c} L\left(\left\{u_{k}\right\},\left\{w_{k}\right\},\lambda \right)=\alpha \sum_{k=1}^{K}{∥\partial_{t}\left[\left(\delta \left(t\right)+\frac{j}{\pi t}\right)u_{k}\left(t\right)\right]e^{-jw_{k}^{t}}∥}_{2}^{2}\\ +{∥f\left(t\right)-\sum_{k=1}^{K}u_{k}\left(t\right)∥}_{2}^{2}+\langle \lambda \left(t\right),f\left(t\right)-\sum_{k=1}^{K}u_{k}\left(t\right)\rangle \end{array}$$

where α denotes the penalty factor, and λ(t) denotes the Lagrangian multiplier.

(3) **Solve the unconstrained variational problem using Fast ADMM**

I. Initialize ${\hat{u_{k}}}^{1}$, ${\omega_{k}}^{1}$, ${\hat{\lambda }}^{1}$, $a^{1}$, n = 0, and k = 1.

II. Update modes ${\hat{u}}_{k}$ and frequencies $\omega_{k}$ using Equations (3) and (4) for each K, and then update ${\hat{\lambda }}^{n}$ using Equation (5).

(3) $$\begin{array}{c} {\hat{u}}_{k}^{n}\left(\omega \right)=\frac{\hat{f}\left(w\right)-\sum_{i\neq k}{\hat{u}}_{i}\left(\omega \right)+\hat{\lambda_{in}}\left(\omega \right)/2}{1+2{\alpha (\omega_{in}-\omega_{k})}^{2}} \end{array}$$

(4) $$\begin{array}{c} \omega_{k}^{n}=\frac{\int \omega \left|{\hat{u}}_{k}{\left(\omega \right)}^{2}\right|d\omega }{\int \left|{\hat{u}}_{k}{\left(\omega \right)}^{2}\right|d\omega } \end{array}$$

Following each iteration, derive the modalities and central frequencies and update the Lagrangian multiplier using the following equation:

(5) $$\begin{array}{c} {\hat{\lambda }}^{n}\left(\omega \right)={\hat{\lambda_{in}}}^{n-1}\left(\omega \right)+\tau \left(\hat{f}\left(\omega \right)-\sum_{k}{\hat{u}}_{k}^{n}\left(\omega \right)\right) \end{array}$$

where τ denotes the update rate.

III. Update Nesterov stepsize $a^{n+1}$ using Equation (6)

(6) $$a^{n+1}=\frac{1+\sqrt{1+4{\left(a^{n}\right)}^{2}}}{2}$$

IV. Update the modalities ${\hat{{u_{in}}_{k}}}^{n}$ and central frequencies ${{\omega_{in}}_{k}}^{n}$ by Equations (7) and (8) for each K, then update the Lagrangian multiplier ${\hat{\lambda_{in}}}^{n}$ by Equation (9).

(7) $$\begin{array}{c} {\hat{u_{in}}}_{k}^{n+1}\left(\omega \right)={\hat{u}}_{k}^{n}\left(\omega \right)+\frac{a^{n}-1}{a^{n+1}}\left({\hat{u}}_{k}^{n}\left(\omega \right)-{\hat{u}}_{k}^{n-1}\left(\omega \right)\right) \end{array}$$

(8) $$\begin{array}{c} {\omega_{in}}_{k}^{n+1}=\omega_{k}^{n}+\frac{a^{n}-1}{a^{n+1}}\left(\omega_{k}^{n}-\omega_{k}^{n-1}\right) \end{array}$$

(9) $$\begin{array}{c} {\hat{\lambda_{in}}}^{n+1}\left(\omega \right)={\hat{\lambda }}^{n}\left(\omega \right)+\frac{a^{n}-1}{a^{n+1}}\left({\hat{\lambda }}^{n}\left(\omega \right)-{\hat{\lambda }}^{n-1}\left(\omega \right)\right) \end{array}$$

V. Evaluate whether the iterative termination condition is satisfied using (10). If it is, the iteration is halted, and the result is produced. Otherwise, increment the iteration number n by 1 and return to Step II.

(10) $$\begin{array}{c} \sum_{k}\frac{{∥{\hat{u_{k}}}^{n+1}-{\hat{u_{k}}}^{n}∥}_{2}^{2}}{{∥{\hat{u_{k}}}^{n}∥}_{2}^{2}}<\varepsilon \end{array}$$

where ε is the discriminant accuracy parameter.

**Figure 2:**
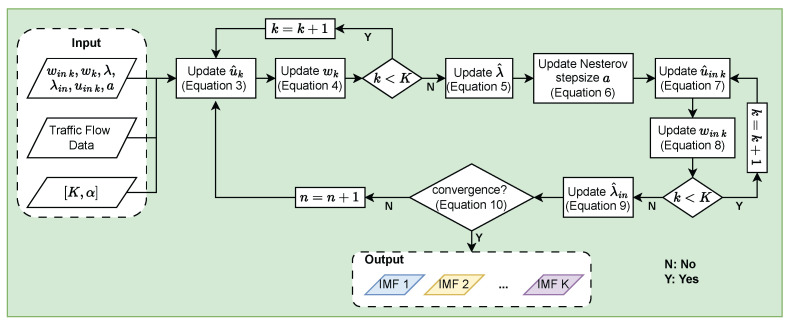
Flowchart of FVMD algorithm.

Figure 2 depicts a flowchart of VMD using fast ADMM.

### 3.3. FVMD Optimized by GA-WOA

**Figure 3:**
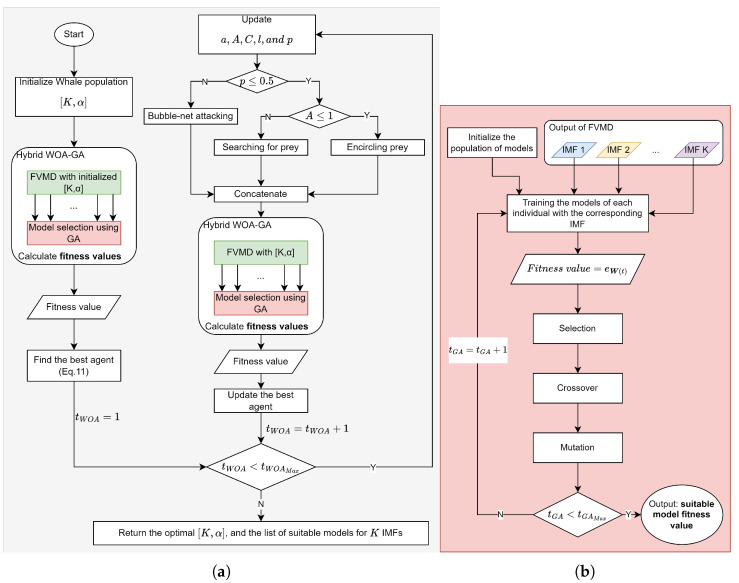
Overview of WOA-GA-optimized FVMD (**a**) WOA-GA; (**b**) GA.

The selection of an appropriate number of decompositions (*K*) and α values is crucial when using FVMD. To address this, we employed WOA, proposed by Mirjalili et al. [37], to refine the FVMD hyperparameters. Following decomposition, each IMF has different characteristics, necessitating the selection of a suitable prediction model. The GA, introduced by Professor J. Holland in 1975, solves this problem by selecting an ML model from a list including the GRU [32], BiGRU [33], LSTM [34], and BiLSTM [35] for each IMF. The combined WOA-GA approach simultaneously ensures optimal parameter tuning and model selection for each IMF. The architecture of WOA-GA-optimized FVMD is illustrated in Figure 3. Specifically, Figure 3a illustrates the WOA-facilitated fine-tuning of model parameters with the aid of the GA for computing the fitness value. The GA-based ML model selection for the calculation is illustrated in Figure 3b. The output of these processes is the optimal combination of hyperparameters with the lowest fitness value, along with the corresponding models. The WOA-GA process can be explained as follows:

I. **Initialization**

To fine-tune FVMD and select an adaptive model for each IMF, we set the total number of iterations for both WOA and GA ($n_{WOA},n_{GA}$) to 10. Additionally, through comprehensive experiments, we determined the optimal number of whales in the WOA and GA populations to be 10. The specific range for each FVMD hyperparameter that requires fine-tuning was determined as follows:

- K∈[2,9]
- α∈[1500,5000]

**Figure 4:**
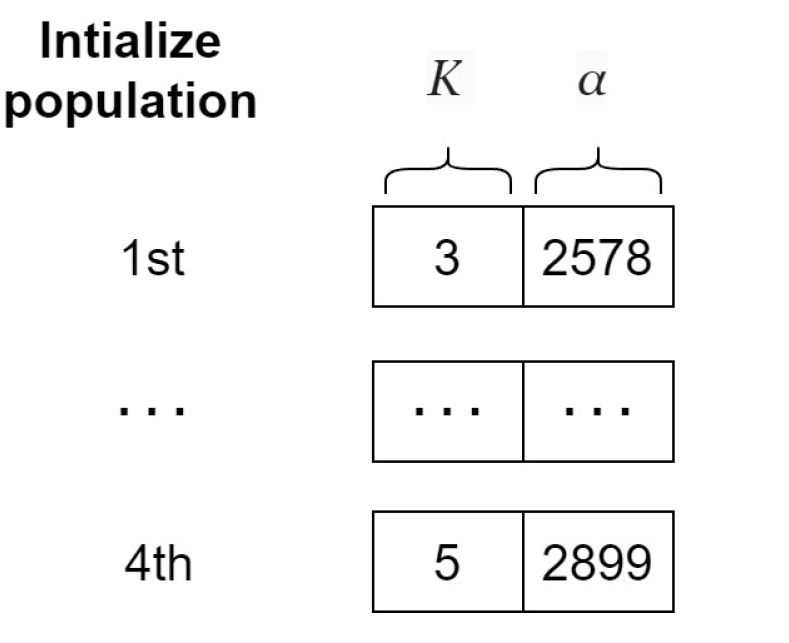
An instance of input WOA population.

First, in Figure 3a, four potential whales are initialized randomly from the input WOA population, as depicted in Figure 4.

II. **Calculating the fitness value**

**Figure 5:**
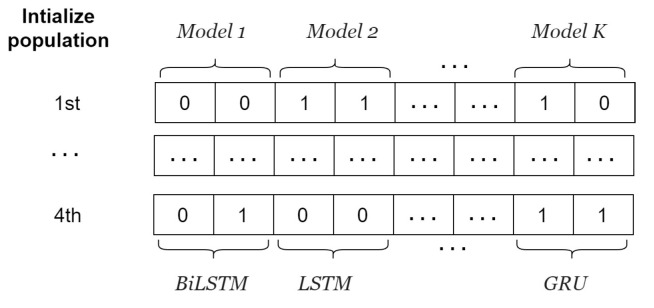
An instance of input GA population for adaptive model selection.

The fitness value is calculated by decomposing the traffic flow data into IMFs using FVMD, as illustrated in Figure 2, with [K,α] derived from the whale population. The outputs of FVMD corresponding to each whale are IMFs. The GA processes these IMFs by selecting suitable models to generate corresponding predictions. The GA population consists of four individuals, each of which has K×2 genes. Specifically, every pair of genes shows the model for each IMF, as illustrated in Figure 5. These lists are then updated using the GA until the most suitable model is obtained. The list of candidate ML models is denoted as follows.

- LSTM: 00
- BiLSTM: 01
- GRU: 11
- BiGRU: 10

**Figure 6:**
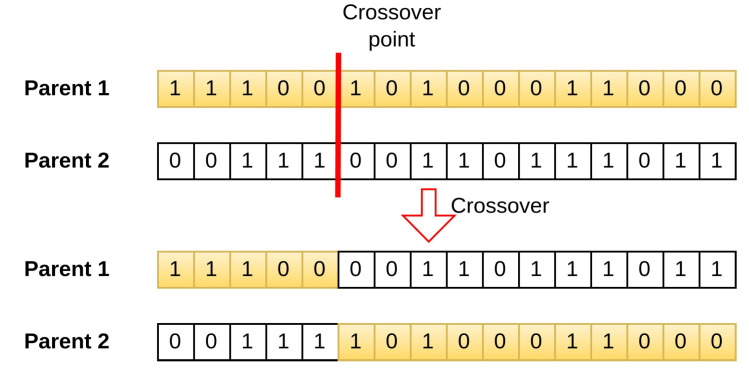
Crossover operator of GA.

The GA involves three main operations: selection, crossover, and mutation. During selection, chromosomes are selected for reproduction based on their fitness, with those with higher fitness assigned a higher chance of becoming parents. These parents then undergo crossover to produce new offspring by exchanging segments of their chromosomes at randomly selected points. Figure 6 illustrates the process.

**Figure 7:**
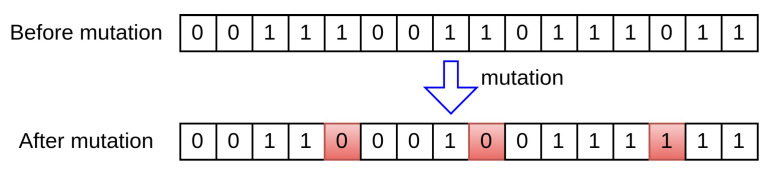
Mutation operator of GA.

Mutation occurs after the crossover operation. This process involves randomly altering one or more genes to generate new offspring, thereby creating new adaptive solutions and avoiding local optima. Specific mutation operations are illustrated in Figure 7. Over multiple generations, the GA converges to the optimal solution.

Following FVMD and GA, each suitable model corresponding to each decomposed IMF is employed as a predictor to create a prediction for its IMF. After that, all predictions are summed up to form the final prediction that is compared with the ground truth using RMSE. The fitness function for both WOA and GA, as defined by Equation (11), is

(11) $$\begin{array}{c} F=e_{\sum_{t=1}^{k}W\left(t\right)} \end{array}$$

where $e_{\sum_{t=1}^{k}W\left(t\right)}$ represents the prediction RMSE (Equation (23)) of all IMFs, and W(·) represents the model selected for each IMF.

With the list of fitness values for the whale population, the best agent is found, initiating a WOA iteration.

III. **Evaluating the iterative termination condition.**

If $t_{WOA}>t_{WOA_{Max}}$, halt the iteration and return the optimal FVMD parameters along with the best-fit model for each corresponding IMF. Otherwise, increment the iteration number $t_{WOA}$ by 1, and update the whale population and WOA parameters.

IV. **Updating the whale population**

In the WOA-GA-optimized FVMD iteration, the whales update their positions using one of three search strategies: surrounding prey, spiral bubble-net predation, or searching for prey. The selection of strategy depends on the values of *p* and |A|, as shown in Figure 3a.

*Surrounding prey*

The optimal position is determined by selecting the location of the whale nearest to the target. Once the optimal position is established, other whales move towards it. The equation for encircling prey behavior is expressed as follows:

(12) $$\begin{array}{c} D=\left|CW_{best}\left(t\right)-W\left(t\right)\right| \end{array}$$

(13) $$\begin{array}{c} W\left(t+1\right)=W_{best}\left(t+1\right)-AD \end{array}$$

where *t* denotes the current iteration, $W_{best}\left(t\right)$ denotes the current optimal position vector of an individual whale, and W(t+1) denotes the next position vector of the whale. The variable D represents the distance between the whale and its prey. The coefficients *A* and *C* are calculated using the following equations:

(14) $$\begin{array}{c} A=2a\times r_{1}-a \end{array}$$

(15) $$\begin{array}{c} C=2r_{2} \end{array}$$

(16) $$\begin{array}{c} a=2-\frac{2t}{n_{WOA}} \end{array}$$

where $r_{1}$ and $r_{2}$ represent random numbers between 0 and 1, the variable *a* decreases linearly from 2 to 0, and $T_{max}$ is the maximum number of iterations.

*Spiral bubble-net predation*

The following mathematical model is used to simulate humpback whale hunting behavior in the form of a spiral:

(17) $$\begin{array}{c} D_{p}=\left|W_{best}\left(t\right)-W\left(t\right)\right| \end{array}$$

(18) $$\begin{array}{c} W\left(t+1\right)=W_{best}\left(t+1\right)-D_{p}e^{bl}\cos\left(2\pi l\right) \end{array}$$

Equation (17) expresses the distance between the prey and the whale *i*th, where the constant variable b is used to define the spiral shape with a value of 1, and *l* is a random value between −1 and 1.

When circling their prey, whales use contracted envelopes and spiral models. A mathematical model can be used to simulate this behavior with a 50% chance of updating positions. The envelope gradually decreases in size during the circling process.

(19) $$\begin{array}{c} W\left(t+1\right)=\left\{\begin{array}{c} W_{best}\left(t+1\right)-AD,p<0.5\\ W_{best}\left(t+1\right)+D_{p}e^{bl}\cos\left(2\pi l\right),p\geq 0.5 \end{array}\right. \end{array}$$

where *p* is a random probability variable between 0 and 1.

*Searching for prey*

When searching for pray, humpback whales swim randomly relative to each other. This behavior can be mathematically modeled as follows:

(20) $$\begin{array}{c} D=\left|CW_{rd}\left(t\right)-W\left(t\right)\right| \end{array}$$

(21) $$\begin{array}{c} W\left(t+1\right)=W_{rd}\left(t\right)-AD \end{array}$$

where $W_{rd}$ are the positions of randomly selected whales. When $\left|A\right|$ is greater than 1, the whales must keep themselves away from their prey, and all whale positions are updated using the randomly generated $W_{rd}$. This strategy is helpful for identifying more appropriate prey species.

After updating the positions of whales, the fitness value calculated in Step II is again applied to determine the optimal whale. These iterative steps minimize the fitness value to determine optimal parameters for FVMD and the best-fit model for each IMF.

V. **Output Selection for GA and WOA**

**Figure 8:**
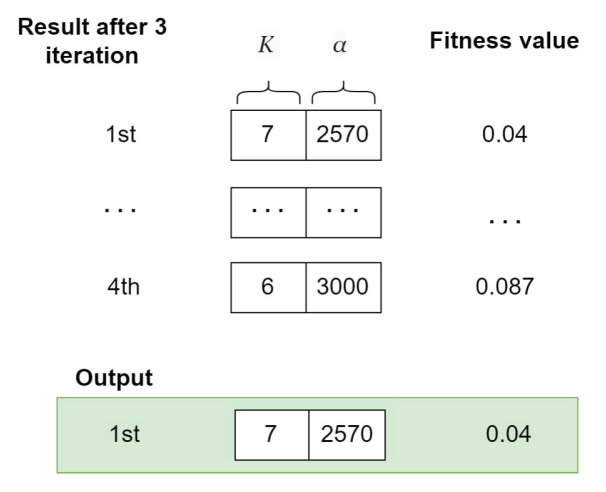
Instance of WOA output.

After four iterations, the whale with the best fitness score is identified, representing the optimal FVMD parameters and a suitable model list for a given dataset. Figure 8 illustrates the selection of an optimal whale.

**Figure 9:**
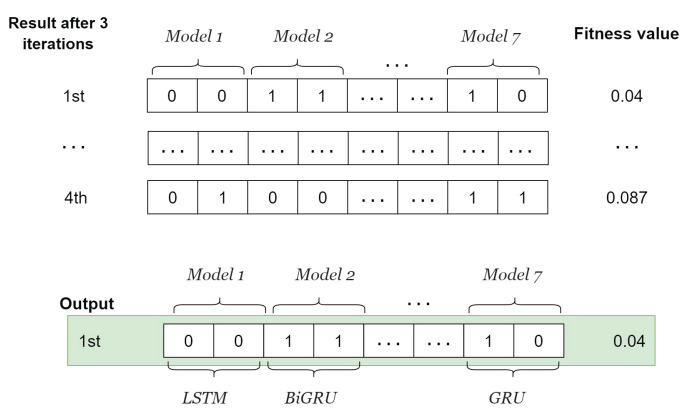
Instance of GA output.

Similarly, the model list with the optimal FVMD parameters and best fitness score is selected as the GA output, as illustrated in Figure 9.

The final output of WOA-GA is the combination of the outputs shown in Figure 8 and Figure 9, representing the optimal FVMD parameters and corresponding optimal model list.

## 4. Experiments and Analysis

### 4.1. Datasets

To evaluate the performance of the proposed method through experiments, two distinct real-life traffic datasets are used: PeMSD08 and the Metro Interstate Traffic Volume dataset.

**Figure 10:**
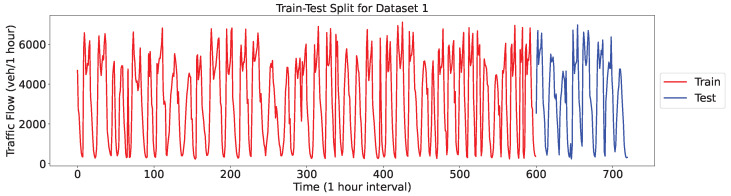
Traffic flow of dataset 1.

The Interstate Traffic Dataset https://archive.ics.uci.edu/dataset/492/metro+interstate+traffic+volume (Dataset 1; accessed on 28 August 2024) provides hourly traffic volume data for the westbound I-94, a major interstate highway in the United States connecting Minneapolis and St. Paul, Minnesota. The Minnesota Department of Transportation (MnDOT) collected the data from 2012 to 2018 at a station midway between the two cities. The dataset encompasses nine features representing traffic volume, holiday status, temperature, and weather. By adopting the same setup as in [26], we randomly sampled 720 continuous data points for our experiments, with 600 used for training and 120 allocated for testing (Figure 10).

**Figure 11:**
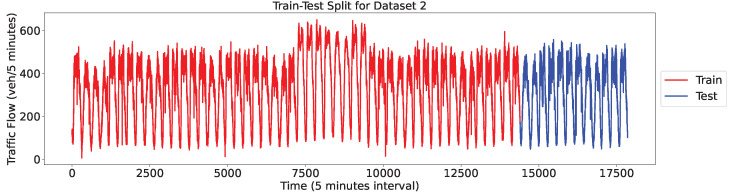
Traffic flow of dataset 2.

The PeMSD dataset contains information on highway traffic in California, collected using the Caltrans Performance Measurement System in real time every 30 s and then aggregated into 5-min intervals. The dataset includes more than 39,000 detectors deployed on highways in major metropolitan areas, along with geographic information about the sensor stations. For our experiment, we used the PEMDS08 [38] variant (Dataset 2) of PEMDS, wherein data were recorded from July to August 2016 using 170 different detectors. We selected 14,400 data points for training (corresponding to 50 days, approximately 80% of the data) and 3456 data points for testing (corresponding to 12 days, approximately 20% of the data), as shown in Figure 11. The detailed statistics information of the two datasets is shown in Table 2.

In traffic flow prediction, a fixed span of past data points—known as a history window—is used to generate predictions about future values. Throughout the experiments, a fixed history window size of 12 was used for both datasets. This means that the models were trained and evaluated using the last 12 time steps of the historical data to predict future values. After being decomposed into IMFs, the data are split into training and testing sets; details of the train/test split are shown in Table 3. Before being fed into the prediction model, the data are normalized using the min–max scaling method to make sure that the data are within the range of [0, 1].

### 4.2. Time Comparison of FVMD and VMD

We investigated the performance of standard VMD and FVMD on the two datasets to compare their running times under varying conditions. During this process, the number of subsequences K was alternated between 2 and 9, and the alpha value was fixed at 2000.

**Figure 12:**
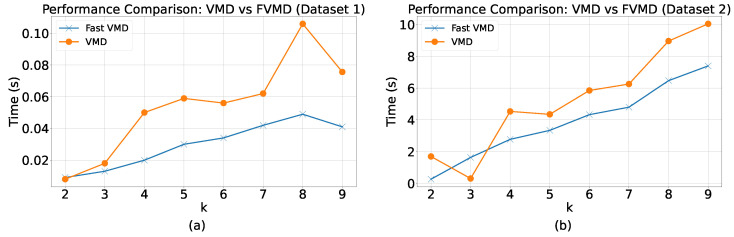
Time Comparison of VMD and FVMD Across Different K Values on Two Datasets. (**a**) Dataset 1, (**b**) Dataset 2.

Figure 12 presents the time consumption of FVMD and VMD under different values of K. For both datasets, the processing time required by both VMD and FVMD increased along with the number of subsequences. As expected, higher K values typically incur a higher computational complexity for the signal decomposition process.

For Dataset 1, the general trend in terms of processing time was better for FVMD than VMD. Given smaller values of K, the two algorithms exhibited similar time costs, as the overall computational complexity of VMD might not be sufficiently high for the adaptive penalty update of fast ADMM to provide a significant advantage over classic ADMM. However, the difference between the two algorithms’ processing times increased alongside K.

For Dataset 2, an exception was observed at K = 3, where FVMD performed slightly worse than VMD, proving that the stability of FVMD is not always maintained. However, K = 3 was not the optimal K for Dataset 2, and the general trend time of FVMD was better than that of VMD.

### 4.3. Comparison Experiment

#### 4.3.1. Evaluation Metrics

To evaluate the prediction results, we used the MSE as the primary metric, along with the RMSE, MAE, and MAPE as references. The following formulas were used:

(22) $$\mathrm{MSE}=\frac{1}{n}\sum_{i=1}^{n}{\left(y_{i}-{\hat{y}}_{i}\right)}^{2}$$

(23) $$\mathrm{RMSE}=\sqrt{\frac{1}{n}\sum_{i=1}^{n}{\left(y_{i}-{\hat{y}}_{i}\right)}^{2}}$$

(24) $$\mathrm{MAE}=\frac{1}{n}\sum_{i=1}^{n}\left|y_{i}-{\hat{y}}_{i}\right|$$

(25) $$\mathrm{MAPE}=\frac{1}{n}\sum_{i=1}^{n}\frac{\left|y_{i}-{\hat{y}}_{i}\right|}{\left|y_{i}\right|}$$

where *n* denotes the number of data points within the test set, $y_{i}$ denotes the ground truth, and $\hat{y}$ denotes the prediction. Because all of these metrics represent error measures, they are inversely correlated with model performance. As an additional evaluation metric, we measured the running time (RT) of each variant.

#### 4.3.2. Baseline Settings and Parameter Configurations

To evaluate the effectiveness of the proposed parameter optimization and adaptive model selection strategy, we compared the performance between various decomposition and prediction configurations across the two datasets. In the proposed method (**FVMD-WOA-GA**), WOA is utilized to select the optimal number of subsequences K and penalty parameter α. Subsequently, FVMD is applied to decompose the time series into IMFs. Finally, the GA is employed to select the most suitable predictor for each IMF subsequence, ensuring that the prediction process is tailored to the characteristics of each component of the signal. For a comprehensive comparison, we set the following baselines:

- **FVMD-WOA-Random**: This variant randomly chooses a predictor for each IMF following the decomposition phase with FVMD (GA not applied).
- **FVMD-WOA-LSTM**: This variant uses LSTM to predict each IMF after the decomposition phase with FVMD (GA not applied).
- **FVMD-WOA-BiLSTM**: This variant uses BiLSTM to predict each IMF after the decomposition phase with FVMD (GA not applied).
- **FVMD-WOA-GRU**: This variant uses GRU to predict each IMF after the decomposition phase with FVMD (GA not applied).
- **FVMD-WOA-BiGRU**: This variant uses BiGRU to predict each IMF after the decomposition phase with FVMD (GA not applied).
- **LSTM**: Simple LSTM model using the original traffic flow as input (without FVMD).
- **BiLSTM**: Simple BiLSTM model using the original traffic flow as input (without FVMD).
- **GRU**: Simple GRU model using the original traffic flow as input (without FVMD).
- **BiGRU**: Simple BiGRU model using the original traffic flow as input (without FVMD).

For simplicity, these variants are referred to as FVMD-WOA-GA #1, FVMD-WOA-Random #2, FVMD-WOA-LSTM #3, FVMD-WOA-BiLSTM #4, FVMD-WOA-GRU #5, FVMD-WOA-BiGRU #6, LSTM #7, BiLSTM #8, GRU #9, and BiGRU #10.

For WOA, the parameter setup included a maximum of four iterations and a population size of 10 whales. The parameter K was constrained to a range of 2 to 9, whereas the parameter α ranged from 1500 to 5000. For the GA, the number of iterations was set to 10 with a population size of 10 and crossover rate of 0.9. For the RNN predictor, the setup included 16 units in the hidden layer, training for 100 epochs with a batch size of 1024, dropout rate of 0.1, and learning rate of 0.01. For FVMD, the parameters were set as follows: tau = 0.0, DC = 0, initialization method = 1, and convergence tolerance = $1\times 10^{-7}$.

#### 4.3.3. Performance with Different History Window Sizes

**Figure 13:**
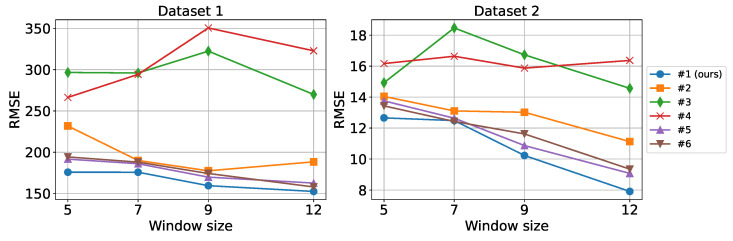
Performance with different history window sizes.

Figure 13 illustrates the performance of different model variants across various history window lengths. We evaluated the model using window lengths of 5, 7, 9, and 12 samples to determine the optimal amount of historical data required for accurate forecasting. As shown in the figure, model performance generally increased along with window size. As expected, the more historical data samples are provided, the more information the model can rely on to produce more accurate predictions. The proposed FVMD-WOA-GA model outperformed the other methods in terms of window size for both datasets. The time interval between two continuous samples in the first dataset is one hour, whereas, in the second dataset, it is five minutes. As a result, the second dataset can retain more information about the non-stationary traffic flow dataset (traffic light stops, congestion, and so on) in a shorter period of time than the first, which maps more general patterns. Therefore, in dataset 2, methods can capture more valuable information, resulting in more improvement when increasing the history window size compared to the first dataset.

#### 4.3.4. Impact of Each Subsequence

**Figure 14:**
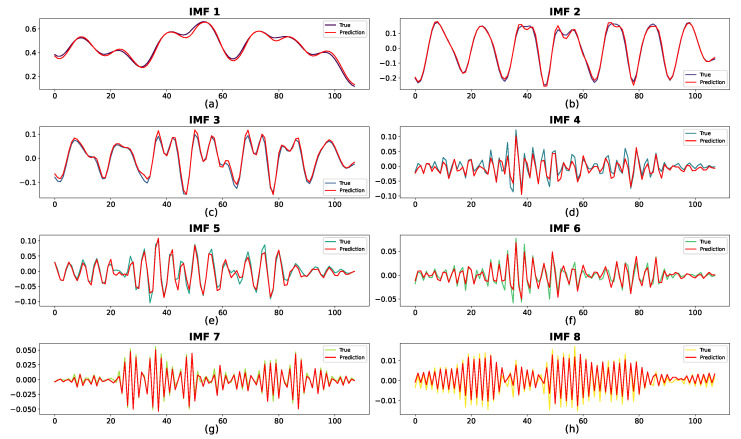
Prediction results of subsequences on Dataset 1 with best suitable K = 8, with corresponding predictors: (**a**) BiGRU, (**b**) BiGRU, (**c**) GRU, (**d**) LSTM, (**e**) LSTM, (**f**) BiLSTM, (**g**) BiGRU, (**h**) BiGRU.

**Figure 15:**
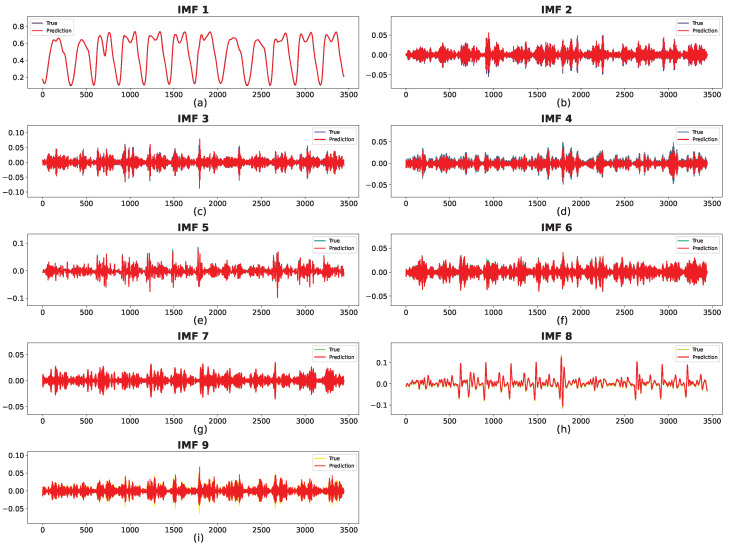
Prediction results of subsequences on Dataset 2 with best suitable K = 9, with corresponding predictors: (**a**) BiGRU, (**b**) BiGRU, (**c**) GRU, (**d**) BiLSTM, (**e**) BiGRU, (**f**) BiGRU, (**g**) GRU, (**h**) BiLSTM, (**i**) BiGRU.

The prediction results for the two datasets are shown in Figure 14 and Figure 15, where the red curve indicates the predicted sequence. The actual curve is completely covered by the red curve, demonstrating the prediction accuracy of the proposed method for each IMF. In Figure 14, despite some bias at the extreme points for IMF4, IMF7, and IMF8, the overall patterns of the predicted and real values are closely aligned. For IMF1–IMF3, the forecasted values are remarkably consistent with the observed trends. In addition, as shown in Figure 15, the predictions for all subsequences are nearly identical to the ground truth.

Ultimately, FVMD was used to efficiently extract and process traffic volume fluctuations in both datasets, significantly enhancing predictive performance. Thus, the combination of FVMD and ML model optimization significantly improved the accuracy of traffic volume prediction.

The FVMD plays a crucial role in decomposing these raw data into multiple simpler sub-signals (IMFs), each capturing different aspects of the traffic flow, such as trends, periodic patterns, and noise. Each IMF requires a tailored predictor to capture its unique characteristics fully and play a different role in the final results. To evaluate their contribution, we sequentially remove each IMF from the final prediction and measure the increase in RMSE. The detailed results are shown in Table 4; in both datasets, the first IMF plays the most crucial role in the final prediction, with a performance loss when not integrating them of approximately 3058.19 and 329.61 in RMSE, corresponding to Datasets 1 and 2. In both datasets, IMFs with a high impact on the final results tend to have a smoother shape in their signal compared to the rest (IMF1 Figure 14a, IMF2 Figure 14b, and IMF3 Figure 14c on Dataset 1; IMF1 Figure 15, IMF8 Figure 15h for Dataset 2). The smoother signal contains the general trend of traffic flow data, resulting in more contribution than the other IMFs, which capture the noise and periodic patterns.

#### 4.3.5. Results of Proposed Method

**Figure 16:**
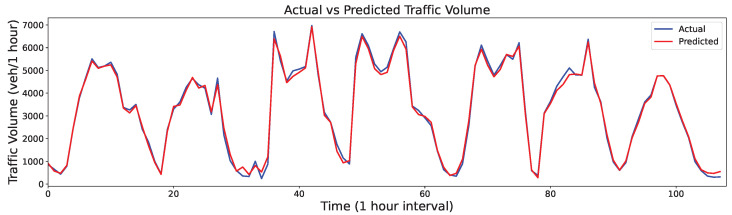
Final prediction results on Dataset 1.

**Figure 17:**
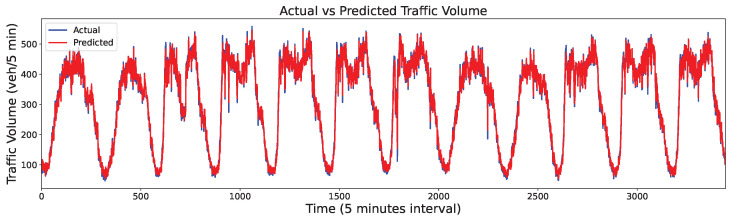
Final prediction results on Dataset 2.

The final traffic volume output value was obtained by integrating the prediction results of all IMFs from Figure 14 and Figure 15, as shown in Figure 16 and Figure 17. Although the predictions exhibited some distortions at extreme points across the two datasets, they generally followed the trends of the actual values. Thus, the proposed FVMD-WOA-GA model demonstrated good prediction accuracy and robustness for both datasets, even when analyzing traffic volume data with dramatic fluctuations.

#### 4.3.6. Analysis of Comparative Results

Table 5 and Table 6 present a comparison of prediction errors measured by five metrics (MAE, MAPE, MSE, RMSE, and RT) on the two datasets (best values for each metric are shown in bold). We compared our proposed approach with four other variants (FVMD-WOA-Random, FVMD-WOA-LSTM, FVMD-WOA-BiLSTM, FVMD-WOA-GRU, and FVMD-WOA-BiGRU) that use only one model to predict traffic flow for all subsequences but still fine-tune the FVMD parameters by WOA.

The results of this experiment show that the FVMD-WOA-GA model consistently outperformed the other variants across all error metrics, indicating its reliability and accuracy in predicting traffic flow. In terms of RMSE, the proposed model demonstrated significant improvements over the second-best-performing configurations across both datasets. For Dataset 1, the proposed model achieved a reduction in RMSE of approximately 3.44% compared with the FVMD-WOA-BiGRU model. On Dataset 2, the proposed model achieved an even greater reduction in RMSE of approximately 12.87% compared to the second-best method (FVMD-WOA-GRU). These findings provide strong evidence for the effectiveness of the proposed model in the field of traffic flow prediction.

In contrast, the configurations that used fixed models for all components had higher error rates, suggesting that the use of a single model for all components might not effectively capture the complexities and characteristics of each subsequence of traffic flow data. Moreover, when predicting traffic flow with just raw signals, the methods have very poor performance with very high RMSE metric compared to the standard deviation of the dataset (variants #6 to #10).

It is worth mentioning that the error rates did not increase uniformly across all models, which can be attributed to the varying effectiveness of different models in capturing traffic flow patterns. For example, the BiLSTM and BiGRU models, which can capture both forward and backward dependencies, achieved lower error rates than the LSTM and GRU models. The GRU and BiGRU models have fewer parameters than the LSTM and BiLSTM models, as they combine the forget and input gates into a single update gate, thereby reducing computational complexity; thus, they exhibit better generalizability and avoid overfitting. The FVMD-WOA-Random configuration, which randomly selects a predictor for each IMF, exhibited relatively inconsistent performance on both datasets.

Owing to the use of GA for predictor selection and WOA for the FVMD parameter selection, the proposed method takes longer to converge, resulting in a longer running time than methods that use a single-type predictor and single models. However, it is essential to note that, in practice, this parameter selection process can be conducted offline to identify the best parameters tailored to the characteristics of the traffic flow data. Once the optimal parameters are determined, the inference phase can be executed in real-time, where the running time is acceptable for practical applications.

To measure the inference time of the proposed methods, we take WOA-GA-integrated variants to predict each sample in the test set and then calculate their average inference time; the results are shown in Table 7. For all WOA-GA-integrated methods, the average inference time is under 10 ms for both datasets. In our experiment, predictors work sequentially. However, in a real 5G environment, if the inference phase can be processed in parallel, the total inference time of the system can be significantly reduced to approximately the average inference time of a single predictor (around 1 ms according to Table 7). These results demonstrate the practicality of the proposed method in real-time environments.

## 5. Conclusions

In this paper, we propose FVMD-WOA-GA as a new method for predicting traffic flow in 5G networks. This method uses FVMD optimized by WOA for parameter optimization, along with GA, to select the best model for each decomposed subsequence. Our results showed that the combination of these models outperformed all other methods on both datasets by effectively capturing different temporal dependencies. Furthermore, the use of fast ADMM improves the efficiency of VMD, reducing the overall time consumption. Our findings highlight the potential of our proposed model to drive accurate and real-time traffic flow predictions, which is crucial for optimizing traffic management and planning in 5G-enabled ITS. By facilitating quicker, more precise decisions, this approach can support dynamic traffic routing, congestion management, and resource allocation in smart cities. Future extensions could include integrating the approach with real-time traffic planning systems on autonomous vehicles, which would enable them to avoid coming to congestion areas and leveraging 5G’s low latency for real-time decision-making to find the optimal route. Additionally, integrating advanced machine learning models, such as Transformer networks, could improve predictive accuracy for complex traffic patterns. We tend to design an encoder of the transformer to serve as FVMD and multiple decoders that are tailored to each feature of each decomposed branch.

## Figures and Tables

**Table 1:** Related works in traffic flow prediction using VMD technique. The “✓” symbol denotes the use of an Adaptive Predictor.

Paper	Year	VMD Fine-Tuning Algorithm	VMD Solving Algorithm	Predictor	Adaptive Predictor
[25]	2021		ADMM	BiLSTM	
ine [26]	2022	cosine similarity	ADMM	ELM	
ine [27]	2022	Fuzzy Entropy	ADMM	Hybrid GAT-BiGRU	
ine [28]	2022		ADMM	Hybrid BiLSTM, GMDH, ELMAN	
ine [29]	2023		ADMM	DELM	
ine [30]	2023		ADMM	LSTM	
ine [31]	2023		ADMM	Auto-correlation	
ine [10]	2023	NGO	ADMM	ARO-KELM	
ine [9]	2023		ADMM	LSTM	
ine [11]	2024	BA	ADMM	L-BILSTM	
ine Our	2024	WOA-GA	Fast ADMM	LSTM, BiLSTM, GRU, BiGRU	✓

**Table 2:** Data characteristics for traffic flow on Dataset 1 and 2.

Dataset	Minimum	Maximum	Average	Std	Unit
1	231	7118	3237.40	2072.45	vehicles per hour
2	6	650	329.84	145.94	vehicles per 5 min

**Table 3:** Description of input-output structure for the training and testing sets in the prediction model.

Subset	Input	Output
Train	$X_{1}^{true},X_{2}^{true},\ldots ,X_{window\_size}^{true}$	$X_{window\_size+1}^{true}$
	$X_{2}^{true},X_{3}^{true},\ldots ,X_{window\_size+1}^{true}$	$X_{window\_size+2}^{true}$
	…	…
Test	$X_{train\_size+1}^{true},X_{train\_size+2}^{true},\ldots ,X_{train\_size+window\_size}^{true}$	$X_{train\_size+window\_size+1}^{true}$
	$X_{train\_size+2}^{true},X_{train\_size+3}^{true},\ldots ,X_{train\_size+window\_size+1}^{true}$	$X_{train\_size+window\_size+2}^{true}$
	…	…

**Table 4:** Impact of removing each IMF on the prediction error (measured in RMSE) for both datasets. The RMSE values of variant #1 (without any IMF removed) are provided in the “None” row. The values in parentheses indicate the increase in RMSE due to the removal of each corresponding IMF. Value of the most impact IMF are shown in bold.

Removed IMF	Dataset 1	Dataset 2
	RMSE	RMSE
None	152.43	7.91
1	**3210.62** (↑ **3058.19**)	**337.52** (↑ **329.61**)
2	954.45 (↑ 802.02)	11.16 (↑ 3.25)
3	498.15 (↑ 345.72)	12.47 (↑ 4.57)
4	268.19 (↑ 115.75)	10.19 (↑ 2.28)
5	300.55 (↑ 148.12)	14.34 (↑ 6.43)
6	237.56 (↑ 85.13)	10.89 (↑ 2.98)
7	234.18 (↑ 81.74)	10.07 (↑ 2.16)
8	163.69 (↑ 11.25)	18.93 (↑ 11.02)
9	-	11.43 (↑ 3.52)

**Table 5:** Comparison results on Dataset 1.

Abbreviation	Model	MAE	MAPE	MSE	RMSE	RT (s)
#1 (ours)	FVMD-WOA-GA	**123.12**	**0.09**	**23,235.66**	**152.43**	432.59
#2	FVMD-WOA-Random	146.23	0.12	35,472.39	188.34	124.21
#3	FVMD-WOA-LSTM	217.87	0.15	72,897.83	270.00	117.49
#4	FVMD-WOA-BiLSTM	249.83	0.18	104,269.15	322.91	99.58
#5	FVMD-WOA-GRU	131.98	0.10	26,434.11	162.59	116.36
#6	FVMD-WOA-BiGRU	124.73	0.11	24,923.34	157.87	122.57
#7	LSTM	689.94	0.44	1,100,158	1048.88	0.282
#8	BiLSTM	702.19	0.45	1,092,327	1045.14	0.29
#9	GRU	713.00	0.45	1,137,029	1066.32	0.30
#10	BiGRU	705.55	0.46	1,096,488	1047.13	**0.278**

**Table 6:** Comparison results on Dataset 2.

Abbreviation	Model	MAE	MAPE	MSE	RMSE	RT (s)
#1 (ours)	FVMD-WOA-GA	**6.17**	**0.027**	**62.55**	**7.91**	2828.57
#2	FVMD-WOA-Random	8.74	0.032	123.79	11.13	1176.37
#3	FVMD-WOA-LSTM	11.21	0.033	212.13	14.56	787.46
#4	FVMD-WOA-BiLSTM	12.67	0.037	267.85	16.37	1108.84
#5	FVMD-WOA-GRU	7.17	0.046	82.36	9.08	914.78
#6	FVMD-WOA-BiGRU	7.38	0.054	87.44	9.35	865.00
#7	LSTM	23.32	0.09	1011.65	31.81	**0.91**
#8	BiLSTM	23.47	0.09	1024.31	32.00	1.18
#9	GRU	22.38	0.08	927.81	30.46	0.93
#10	BiGRU	21.98	0.08	891.48	29.86	0.92

**Table 7:** Average inference times of different methods across two datasets. “K” represents the best number of IMFs selected for each model. At the same time, “Avg” shows the average inference time (in ms) per sample in the test set, and “Per Predictor” indicates the average inference time for each predictor (in ms).

Abbreviation	Model	Dataset 1			Dataset 2		
		K	Avg (ms)	Per Predictor (ms)	K	Avg (ms)	Per Predictor (ms)
#1 (ours)	FVMD-WOA-GA	8	8.20	1.03	9	7.87	0.87
#2	FVMD-WOA-Random	8	7.81	0.98	9	8.11	0.90
#3	FVMD-WOA-LSTM	9	9.34	1.04	5	5.03	1.01
#4	VMD-WOA-BiLSTM	4	4.20	1.05	8	7.74	0.97
#5	FVMD-WOA-GRU	8	7.28	0.91	7	6.01	0.86
#6	FVMD-WOA-BiGRU	8	8.03	1.00	8	7.06	0.88

## Data Availability

Suggested Data Availability Statements are available in Section 4.1.
